# The response of head and neck squamous cell carcinoma to cetuximab treatment depends on Aurora kinase A polymorphism

**DOI:** 10.18632/oncotarget.2117

**Published:** 2014-06-18

**Authors:** Anja Pickhard, Michael Siegl, Alexander Baumann, Maximilian Huhn, Markus Wirth, Rudolf Reiter, Martina Rudelius, Guido Piontek, Gero Brockhoff

**Affiliations:** ^1^ Department of Otolaryngology Head and Neck Surgery, Technical University of Munich, Muenchen, Germany; ^2^ Department of Otolaryngology Head and Neck Surgery, Section of Phoniatrics and Pedaudiology, University of Ulm, Ulm, Germany; ^3^ Institute of Pathology, Julius-Maximilians-University and Comprehensive Cancer Center Mainfranken, Wuerzburg, Germany; ^4^ Department of Gynecology and Obstetrics; University of Regensburg, Regensburg, Germany

**Keywords:** Aurora kinase A polymorphism, Aurora kinase B, cetuximab, HNSCC

## Abstract

**Objectives:**

The aim of this study was to evaluate the efficiency of cetuximab-based anti-EGFR treatment and Aurora kinase A / B knockdown as a function of Aurora kinase polymorphism in HNSCC cell lines.

**Materials and methods:**

First, protein expression of Aurora kinase A / B and EGFR and Aurora kinase A polymorphism were studied in tumour samples.

The survival and proliferation of Aurora kinase A homo- (Cal27) and heterozygous (HN) HNSCC cell lines was evaluated using a colony formation assay and a flow cytometric assay. Also, aneuploidy was determined. EGFR signalling pathway were visualised by western blotting.

**Results:**

Immunohistochemistry revealed the overexpression of Aurora kinase A / B in HNSCC. The knockdown of each kinase caused a significant decrease in clonogenic survival, independent of Aurora kinase A polymorphism. In contrast, cetuximab treatment impaired clonogenic survival only in the Aurora kinase A-homozygous cell line (Cal27).

**Conclusion:**

This study provides in vitro evidence for the predictive value of Aurora kinase A polymorphism in the efficiency of cetuximab treatment. Resistance to cetuximab treatment can be overcome by simultaneous Aurora kinase A/B knockdown.

## INTRODUCTION

Head and neck squamous cell carcinoma (HNSCC) is the sixth most common cancer worldwide [1]. Despite recent advances in surgery and adjuvant treatment options, an overall cure is only achieved in less than 50% of cases. In contrast to many other malignant diseases, HNSCC rarely shows distant metastases at the time of diagnosis but shows a higher incidence of systemic spread [2]. Furthermore, patients with recurrent or metastatic disease have a poor prognosis, with a mean survival of 6-10 months [2].

The overexpression of epidermal growth factor receptor (EGFR) in HNSCC is often caused by gene amplification [3], and this elevated expression is associated with a poor disease outcome and an increased risk of metastasis [4, 5]. The extension of the standard first-line therapy regimen of cisplatin/5-fluorouracil with tumour treatment using the monoclonal antibody C225 (cetuximab) has increased the rate of objective responses and also improved the progression-free (PFS) and overall survival (OS) of patients with progressive / metastatic HNSCC [6, 7]. Nevertheless, the individual patient response to this target specific treatment varies significantly and is not predictable.

DNA gains on chromosome 20q are frequently found in HNSCC [8, 9] and are associated with lymph node metastasis, as shown by array-based comparative genomic hybridisation [10]. The Aurora kinase A (AurkA) encoding region is located on 20q13.2 and maps close to the critical region of DNA gain [11]. Aurora kinases A and B (AurkA and AurkB) are highly conserved serine/threonine kinases that play essential but distinct roles in mitosis. Specifically, AurkA is required for the assembly of the mitotic spindle and accumulates on centrosomes at the spindle poles during prophase until metaphase [12]. Furthermore, the up-regulation of AurkA leads to abnormal centrosome numbers and the induction of aneuploidy [13, 14], which is a very frequent event in HNSCC and found in up to 90% of all tumours [15]. In contrast, AurkB is required for mitotic progression and cytokinesis and colocalises with inner centromeric protein (INCENP) and survivin at the centromeres of chromosomes during cytokinesis. AurkB is involved in chromosome alignment at the spindle midzone during the metaphase / anaphase transition [12, 16]. A T91A polymorphism in AurkA/STK15 causes a Phe31Ile substitution, and the 31Ile variant has been shown to be preferentially amplified and is associated with the degree of aneuploidy in human tumours. AurkA polymorphism has also been described as a genetic risk factor for the occurrence and progression of oesophageal tumours [17]. Moreover, a multiple cancer type meta-analysis revealed a significant cancer risk in both homozygotes and heterozygotes [18].

Recently, we found that patients characterised by elevated EGFR and elevated AurkA protein expression in tumour tissue represent a risk group with a poor disease-free survival [19]. AurkA and EGFR share downstream signalling pathways, and each by itself represents a potential therapeutic target in HNSCC. In the present study, we determined the distribution of AurkA/STK15 codon 91 homo- and heterozygosity in primary HNSCC and the associated non-neoplastic squamous epithelium. Moreover, we analysed the response to cetuximab and the inhibition of AurkA and AurkB *in vitro* according to AurkA/STK15 polymorphism.

## RESULTS

### Elevated AurkA/B expression in HNSCC tissues

**Fig. 1:**
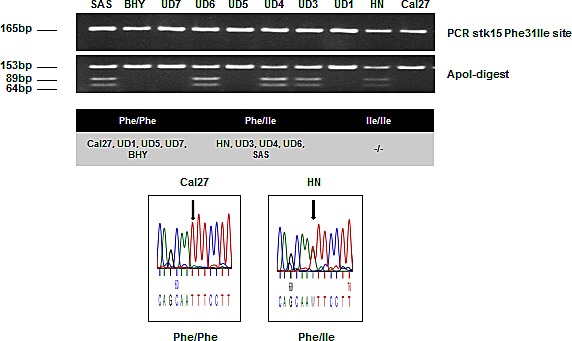
AurkA/STK15 Phe31Ile polymorphism analysis by PCR-RFLP and subsequent DNA sequencing A total of 10 cell lines were tested and showed 50% Phe/Phe, 50% Phe/Ile, and 0% Ile/Ile.

Immunohistochemical staining of HNSCC tissue revealed the overexpression of AurkA and AurkB compared to the corresponding healthy tissue (p < 0.05). The distribution of AurkA/STK15 codon 91 homo- and heterozygosity in the normal (n=64), non-neoplastic tissue of tumour patients (n=41) and tumour tissue (n=116) was determined by a restriction analysis of amplified AurkA/STK15 cDNA. The heterozygous allele was found in 37% and 33% of the normal and non-neoplastic tissues, respectively, whereas the portion increased to 49% in the tumour tissue (Suppl. Fig. 1). In addition, 10 HNSCC cell lines were analysed for the polymorphism, and a 50/50 distribution was observed (Fig. 1). A heterozygous (HN) and homozygous wildtype (Cal27) HNSCC line were selected for further *in vitro* experiments; the genotype of codon 91 in these cell lines was verified by sequencing (Fig. 1).

### Cetuximab treatment impairs AurkA/STK15 codon 91 polymorphism-dependent clonogenic survival

**Fig. 2:**
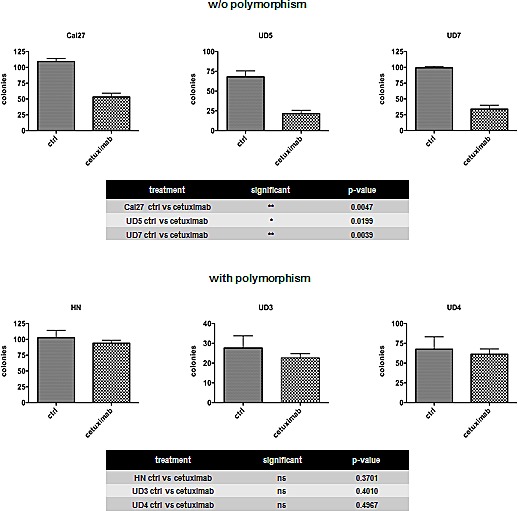
Resistance to cetuximab was associated with AurkA/STK15 Phe31Ile polymorphism (UD3, UD4, and HN) The cell lines UD5, UD7, and Cal27, which are homozygous wildtype for Phe31, exhibited a significant decrease in clonogenic survival.

It has been previously shown that cetuximab is a potent drug for the treatment of HNSCC [20, 21]. In the present study, we tested 6 HNSCC lines for their susceptibility to cetuximab treatment. The Cal27, UD5, and UD7 cell lines showed a dramatic decrease in clonogenic survival after treatment, whereas the HN, UD3, and UD4 cells appeared to be resistant to cetuximab (Fig. 2). Resistance to cetuximab treatment has been associated with the AurkA/STK15 Phe31Ile polymorphism. In contrast to the UD3, UD4, and HN cells, which harbour the polymorphism and did not respond to cetuximab treatment, the Phe31 homozygous wildtype UD5, UD7, and Cal27 cells (UD5 p = 0.0199; UD7 p = 0.0039; Cal27 p = 0.0047) showed a significant decrease in clonogenic survival with antibody treatment.

### siRNA-mediated Aurora kinase A / B knockdown impairs clonogenic survival, independent of polymorphism

**Fig. 3:**
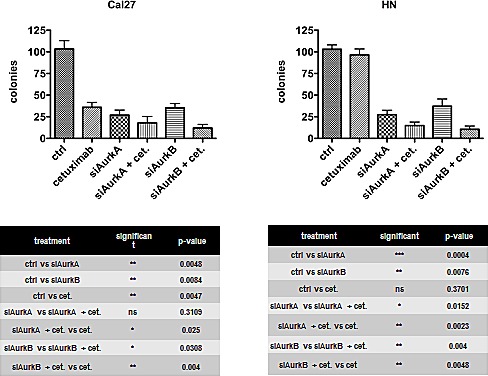
The knockdown of each kinase caused a drastic and highly significant decrease in clonogenic survival The same effect was observed in HN cells, though this cell line did not response to cetuximab alone.

It has been shown that the inhibition of Aurora kinases overcomes resistance to cetuximab in HNSCC [19]. Therefore, we knocked down the expression of these kinases by treating the cells with an AurkA- or AurkB-specific small interfering RNA (siRNA). The siRNA-mediated knockdown of either AurkA or AurkB was highly effective (Suppl. Fig. 2) and specific; furthermore, the knockdown of AurkA did not affect the AurkB protein content and vice versa. The knockdown of each kinase caused a drastic and highly significant decrease in clonogenic survival (Fig. 3), an effect that was independent of AurkA polymorphism (Cal27 - siAurkA p = 0.0048, siAurkB p = 0.0084; HN - siAurkA p = 0.0004, siAurkB p = 0.0076). Treatment with the EGFR inhibitory antibody cetuximab also impaired clonogenic survival in the AurkA/STK15 Phe31Ile polymorphism-negative cell line Cal27 (p = 0.0047). Conversely, the HN cell line, which harbours the polymorphism, was resistant to cetuximab treatment with regard to clonogenic survival. To test the effect of the combined targeting of Aurora kinases and EGFR, both cetuximab and the AurkA/B-specific siRNAs were applied, resulting in a further impairment of clonogenic survival in the Cal27 cells compared to the treatment with cetuximab alone. The combination treatment was also more effective than the knockdown alone, and the combination effect was even significantly increased with AurkB knockdown. The same effect was observed in the HN cells, though this cell line did not respond to cetuximab treatment alone (Fig. 3).

### Induction of aneuploidy in HN cells upon AurkB knockdown but not by cetuximab treatment

**Fig. 4:**
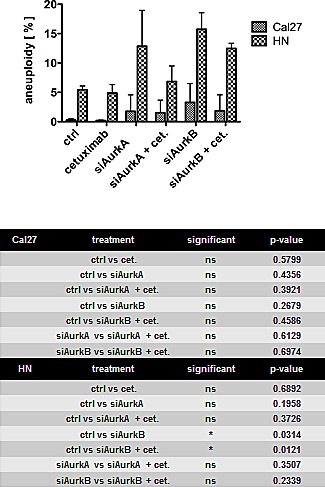
Treatment caused aneuploidy only in the HN cell line with the Phe31Ile polymorphism The knockdown of AurkB had a significant impact on ploidy, whereas cetuximab treatment had no effect on ploidy.

Because Aurora kinases are important for the proper assembly of the spindle apparatus and chromosome segregation in mitosis, the effect of Aurora kinase knockdown on the ploidy of the cells was tested by flow cytometry. Aurk/B knockdown significantly resulted in 15% aneuploid HN cells (p = 0.0314), which harbour the Phe31Ile polymorphism, whereas cetuximab treatment did not affect ploidy (Fig. 4).

### Combined treatment of cetuximab and Aurora kinase knockdown increases apoptosis

**Fig. 5:**
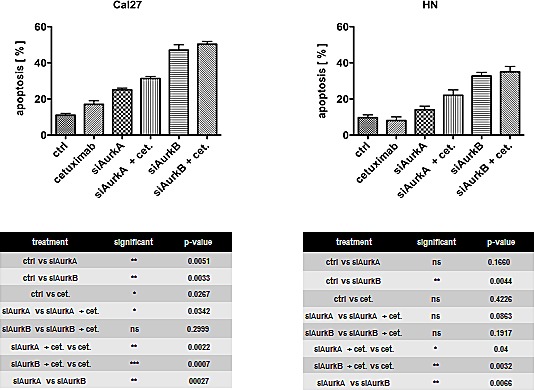
Apoptosis was detectable by flow cytometry after the knockdown of the respective Aurora kinase The knockdown of AurkB led to a significantly higher number of apoptotic cells than AurkA suppression.

Apoptosis was detectable by flow cytometry after the knockdown of the respective Aurora kinase (Fig. 5). The knockdown of AurkB led to a significantly higher amount of apoptotic cells than the suppression of AurkA (Cal27 p = 0.0027; HN p = 0.0066). The Cal27 cells showed a trend of a higher amount of apoptotic cells upon treatments, though cetuximab treatment alone induced only a small but significant amount of apoptosis in these cells (p = 0.0267). This finding is in agreement with the decreased clonogenic survival in this cell line upon cetuximab treatment. The number of apoptotic cells could be further increased with high significance when combined with Aurora kinase knockdown. However, compared to the number of apoptotic cells already induced by the Aurora kinase knockdown, this increase was not significant, except for AurkA knockdown in Cal27 cells (p = 0.0342). This observation indicates a higher importance of Aurora kinase knockdown, particularly AurkB, with respect to cell survival / cell death.

### Treatment with siRNA targeting AurkB causes enhanced apoptosis by reducing Akt activity

**Fig. 6:**
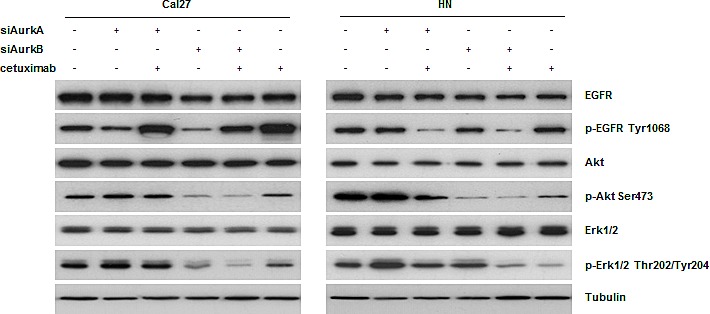
According to western blot analyses, cetuximab treatment resulted in Tyr1068 phosphorylation of EGFR in cetuximab-sensitive Cal27 cells Moreover, we found a downregulation of Akt after treatment with an siRNA against AurkB in both cell lines.

To identify the proteins involved in the regulation and control of cell proliferation and survival, a western blot analysis was performed after AurkA / AurkB siRNA and cetuximab treatments and their combination (Fig. 6, Suppl. Fig. 3). As the activity of these proteins is typically regulated by posttranslational modifications, phosphorylation-specific antibodies were used. The EGFR, Akt, and Erk1/2 expression levels were not affected by any of the treatments. In the Cal27 but not HN cells, cetuximab resulted in a drastic increase in the phosphorylation of its target, EGFR, at Tyr1068. Akt phosphorylation at Ser473 was significantly decreased upon AurkB knockdown in both cell lines; AurkB knockdown alone and in combination with cetuximab treatment resulted in an identical Akt phosphorylation state.

Erk1/2 (a member of the MAP kinase family) phosphorylation at Thr202/Tyr204 was decreased upon AurkB knockdown in the Cal27 cells, in both the presence and absence of cetuximab. In the HN cells, the knockdown of each Aurora kinase caused a slight increase in Erk1/2 phosphorylation, whereas the combination treatment decreased the amount of phosphorylated Erk1/2.

## DISCUSSION

HNSCC is the sixth most common cancer worldwide [1] and typically has a poor prognosis. Unfortunately, new therapeutic regimens, e.g., the combination of standard (chemo-) therapies with cetuximab treatment, merely prolong the overall survival by a few months [20], and therapy response to cetuximab treatment can only be insufficiently evaluated by cutaneous side effects [22, 23]. Because Aurora kinases are frequently upregulated in HNSCC and EGFR is often expressed, both of these molecules represent attractive therapeutic targets. In this study, we investigated the role of Aurora kinases A and B on tumour cell survival and apoptosis in HNSCC *in vitro*. Furthermore, we examined the impact of AurkA polymorphism on the susceptibility of HNSCC to cetuximab treatment.

We confirmed the observation that the AurkA and AurkB proteins are significantly overexpressed in HNSCC tissues [24-29], and it has been demonstrated that an upregulation of AurkA mRNA and protein in HNSCC is associated with a poor outcome [30]. Moreover, it has been shown that AurkA overexpression and/or dysfunction results in chromosomal instability through abnormal spindle formation and defects in cytokinesis, resulting in aneuploidy [31-34]. Accordingly, these genomically altered cells can promote tumour progression, with a poor disease outcome. We previously demonstrated that a subgroup of HNSCC patients with reduced DFS and a particularly poor prognosis is characterised by concomitant AurkA and EGFR overexpression [19].

Our RFLP analysis revealed the distribution of AurkA polymorphism in HNSCC specimens (48% Phe/Phe, 49% Phe/Ile, and 3% Ile/Ile) and normal tissues (67% Phe/Phe, 33% Phe/Ile, and 0% Ile/Ile). Although the Phe/Phe polymorphism is common and the Phe/Ile variant is more frequently found in tumour tissues, no correlation of either variant with the course or outcome of the disease could be found. Nevertheless, the AurkA polymorphism has been observed in many tumour types, e.g., oesophageal, breast, hepatocellular, and prostate cancer [17, 35-38]. Miao et al. found a 44.2% frequency of the heterozygous allele in oesophageal tumour patients, the same range as found in the present study (49%). In contrast, the frequency of the Ile/Ile polymorphism found in other tissues was clearly higher at 47.0% [17]. The prognostic value of either polymorphism, however, is unclear and remains controversial [17, 35-38].

Although we found a reduced capacity to form cell colonies in the presence of the Phe/Ile polymorphism, which was associated with cetuximab resistance, the clinical studies to date do not suggest any correlation between STK15/AurkA polymorphism and cetuximab treatment resistance. Astsaturov et al., however, identified EGFR downstream signalling molecules involved in the resistance to cetuximab treatment. These authors observed synergistic treatment efficiency by a pronounced decrease in cell viability and tumour size upon simultaneous AurkA and EGFR targeting, revealing evidence for AurkA / EGFR signalling crosstalk [39]. In addition, an increased rate of relapse, a poorer response rate, and shortened disease-free and median survival rates have been reported for oesophageal carcinoma patients with the Phe/Ile polymorphism who were treated with chemo-radiotherapy followed by surgery [40]. Hence, there is accumulating evidence for the involvement of the heterozygous Aurora kinase polymorphism Phe/Ile in disease outcome and anti-EGFR/anti-Aurora kinase treatment susceptibility. Although, we connected data which were collected by the retrospective analysis of (primary) tissues taken ex-vivo and data derived from well characterized HNSCC cell lines our findings need to be corroborated by prospective clinical trials.

Our analyses of clonogenic survival revealed that the STK15/AurkA homozygous Cal27, UD5, and UD7 cells efficiently respond to cetuximab mono-treatment, whereas the heterozygous HN, UD3, and UD4 cells do not. The homozygous Cal27 and heterozygous HN cells exhibited a decreased clonogenic survival after the knockdown of both AurkA and AurkB, and apoptosis was largely induced in the Cal27 cells by all the treatment regimens against EGFR or AurkA / B. The efficiency of the combined treatment was additive in the Cal27 cells, and, even more importantly, cetuximab resistance in the HN cells was abolished by AurkA / B knockdown. The overall efficiency of the combined anti-EGFR/anti-Aurora kinase treatment reached a similar level in the heterozygous and homozygous cell lines. The enhanced treatment efficiency can possibly be explained by the cetuximab-related inhibition of EGFR/STAT5A interactions, which intensified the siRNA-mediated knockdown of AurkA expression by the cetuximab functional knockdown of any remaining AurkA protein [41]. The heterozygous HN cells but not homozygous Cal27 cells developed aneuploidy after AurkA / B knockdown, a phenomenon that suggests at least in part different response mechanisms, which can likely be attributed to AurkA polymorphism and a greater degree of chromosomal instability in HN cells.

In the Cal27 cells, we found that cetuximab treatment induced Tyr1068 EGFR-phosphorylation, which has been previously associated with treatment response [42]. Regardless of EGFR phosphorylation, both the Cal27 and HN cells showed reduced Akt activation upon AurkB knockdown that strongly correlated with reduced cell survival. The AurkA knockdown, however, did not affect the EGFR downstream survival pathway. In addition, pan-inhibition of Aurora kinases by cell treatment with VX-680, causes pronounced AurkB inhibition [43], suppresses Akt-1 activation and inhibits cell proliferation [44]. Thus, the MAPK-related proliferation pathway was only affected by the combined treatment with siRNA against AurkB (but not AurkA) and cetuximab. Sharma et al. demonstrated that AurkB is a downstream target of B-raf in melanoma and inhibition of AurkB lead to a reduced tumor development [45]. These findings could explain the outcome of combined AurkB targeting.

Taken together, the data indicate that both the EGFR and Aurora kinases seem to play important roles in the tumour biology and treatment of HNSCC. Further knowledge of the molecular mechanisms underlying simultaneous EGFR / Aurora kinase targeting will facilitate the design of the most efficient therapeutic strategies in consideration of AurkA polymorphism.

## Conclusion

Thus far, the response to cetuximab-based anti-EGFR therapy can only be estimated by the degree of skin reactions; markers that can reliably predict therapy efficiency have not yet been described. Here, we provide evidence that AurkA genotypically homozygous HNSCC cells respond to cetuximab mono-treatment, whereas heterozygous cells do not. Moreover, cetuximab resistance can be overcome by siRNA-based AurkA / B knockdown *in vitro*. The combination of cetuximab and anti-AurkA/B targeting in HNSCC cells ameliorates any polymorphism-related difference and increases the treatment efficiency, independent of the Aurora kinase genotype. However, the efficiency of such a therapy as a function of AurkA polymorphism remains to be investigated more extensively in a clinical setting. A translational approach will enable the identification of patients who may potentially benefit from cetuximab treatment alone and those who require additional modular targeting.

## MATERIAL AND METHODS

### Tissue samples

Formalin-fixed and paraffin-embedded (FFPE) tumour samples from 264 patients with a squamous cell carcinoma of the oral cavity (n=42), oropharynx (n=101), hypopharynx (n=49), or larynx (n=72) were examined for AurkA and AurkB expression. The expression levels were compared to the non-cancerous tissues from some of the same patients (matched pairs). This study was approved by the Medical Ethics Committee of the Technical University of Munich (project number 1420/05).

### Tissue microarray preparation

Core needle biopsies from representative areas of each tumour specimen were retrieved from the original tumour blocks using a manual array (Beecher Instruments, Sun Prairie, Wisconsin, USA) and placed in a recipient paraffin array block; we obtained at least three tissue cylinders, with a diameter of 0.6 mm, from each biopsy specimen.

### Tissue microarrays and protein analyses

An HNSCC tissue microarray (TMA) containing tumour tissues with representative areas of interest and non-neoplastic squamous epithelium was used for the protein analyses. The TMA was sectioned, placed on coated glass slides, and deparaffinised for the subsequent procedures.

### Immunohistochemical study

Fresh 1.5 μm sections from the TMA blocks were transferred to glass slides, deparaffinised, and rehydrated. For antigen retrieval, the slides were heated in citrate-buffered saline (pH=6), and the slides were incubated for 1 hour with antibodies against Aurora kinase A (rabbit, clone D38B1, 1:200; Cell Signaling Technology, Frankfurt, Germany) and Aurora kinase B (rabbit, 1:200; Cell Signalling Technology, Frankfurt, Germany). The reaction was developed using the labelled streptavidin-biotin-peroxidase system. After counterstaining with haematoxylin, the slides were dehydrated in ascending ethanol concentrations and mounted. Tissues with known expression of the respective antigen were used as positive controls; immunoglobulin isotypes were used as negative controls.

The staining intensity and patterns were rated by the following scoring system for the semiquantitative estimation of expression levels: from 0 to 3 points (0 points = no staining, 1 point = low staining intensity, 2 points = moderate staining intensity, 3 points = strong staining intensity). Additionally, the fraction of stained cells was determined and graded from 0 to 4 points (0 points = 0% of the tumour cells, 1 points = <10% of the tumour cells, 2 points = 10%-29% of the tumour cells, 3 points = 30%-59% of the tumour cells, 4 points = 60%-100% of the tumour cells). Both the staining intensity and fraction of stained cells were added together to generate a score from 0 to 7.

### AurkA/STK15 polymorphism analyses: PCR-restriction fragment length polymorphism (RFLP)

DNA was isolated from cell lines (n=10) and the FFPE tumour (n=116) and control (n=64) samples using the DNeasy Kit (Qiagen, Hilden, Germany) according to the manufacturer's instructions.

The specific PCR for AurkA genotypes at the Phe31Ile site was performed using the Taq-DNA Polymerase all-inclusive Kit (PeqLab, Erlangen, Germany) according the manufacturer's guidelines. We applied the following thermocycler (BioRad, Munich, Germany) PCR programme: (95°C for 5 min, 30 cycles of 95°C for 30 s, 60°C annealing for 30 s, and 72°C for 30 s), and 72°C for 7 min. The following primers were used: AurkA/STK15 forward, CTTTCATGAATGCCAGAAAGTT, and reverse, CTGGGAAGAATTTGAAGGACA.

The 165-bp PCR products were digested with ApoI (New England BioLabs, Inc., Beverly, USA) and separated on a 2.5% agarose gel. Digestion of the AurkA/STK15 31Phe allele results in two fragments (153 bp and 12 bp), whereas the AurkA/STK15 31Ile allele contains an additional third ApoI restriction site and thus three bands (89 bp, 64 bp, and 12bp) are generated after digestion. The results were further confirmed by DNA sequencing using an ABI 3100 DNA sequencer (Life Technologies, Darmstadt, Germany).

### Cell culture

The Cal27 and HN cell lines were obtained from DSMZ (Braunschweig, Germany), and the UD3, UD4, UD5, and UD7 cell lines were obtained from the University of Düsseldorf. The AurkA polymorphism status of the cell lines is shown in Fig. 1. Additionally, all cell lines present a moderate expression of EGFR by western blot analyses. The cells were cultured in Dulbecco's Modified Eagle Medium (DMEM) (Invitrogen, Darmstadt, Germany) containing 10% foetal calf serum (FBS) (Biochrom, Berlin, Germany), 2 mM glutamine, 100 μg/ml streptomycin, and 100 U/ml penicillin (Biochrom, Berlin, Germany), maintained at 37 °C in an atmosphere of 5% CO_2_, and grown to 70-90% confluence.

### RNA interference

Cal27 and HN cells were seeded in 6-well tissue culture dishes in a medium containing serum and antibiotics. Shortly before transfection, the medium was changed to OptiMEM. A mixture of 4 μl Oligofectamine™ Transfection Reagent (Invitrogen, Frankfurt, Germany) and 30 nM Stealth RNAi™ siRNA duplex oligoribonucleotides in OptiMEM was added, and the cells were incubated for 48 hours before seeding for further investigation. siRNA duplex oligoribonucleotides targeting AurkA (primer AURKAHSS1861148) and AurkB (primer AURKBHSS90047) were obtained from Invitrogen. The knockdown efficiency was monitored by western blotting.

### Inhibitors

The mouse-human chimeric EGFR-inhibitory antibody cetuximab was purchased from Merck, Darmstadt, and 140 nM cetuximab was used to treat the cells.

### Colony formation assay

Cell survival was assessed by a colony formation assay. The cells were seeded in 6-well tissue culture dishes (5 × 10^2^ cells/well); after 1 d, the Aurora A / B-knockdown cells and control cells were treated with cetuximab (140 nM) and cultured. After 10 days, the cell colonies were subsequently formalin fixed and visualised by crystal violet staining (Sigma-Aldrich, Steinheim, Germany); the colonies were counted manually.

### Flow cytometry

All flow cytometric analyses were performed using the FACSCanto-II flow cytometer (BD Biosciences, San Jose, CA, USA) equipped with a blue (488 nm), a red (633 nm), and a violet (405 nm) laser and a standard optical configuration. A 530/30 bandpass filter, a 670-nm longpass filter, and a 450/50 bandpass filter were installed for green (FITC), red (PI), and blue (Dapi) fluorescence detection, respectively. The data acquisition and analysis were performed with the FACSDiva software (Ver. 6.1.1, BD Biosciences, San Jose, CA, USA).

### Flow cytometric quantification of apoptosis

An AnnexinV/propidium iodide (PI) assay was employed to quantify the induction of apoptosis by the individual treatment (cetuximab, siAurkA, siAurkB, and combinations). This assay enabled the quantification of vital (AnnexinV/PI negative), early (AnnexinV positive/PI negative), and late apoptotic (AnnexinV/PI double positive) cells at each measurement. The cells were seeded, incubated, and treated as described above. After 24 hours and 48 hours growth intervals, the cells were harvested by trypsinisation, and the cell culture supernatant (potential dead and detached cells) of each sample was collected and pooled with the harvested cells. Untreated cells served as a negative control. The cells were immediately stained after harvesting using the TACS™ AnnexinV-FITC Apoptosis Detection Kit (R&D Systems, Wiesbaden-Nordenstadt, Germany) according to the manufacturer's instructions. Single-colour controls (unlabelled, AnnexinV-FITC-labelled, and PI only-labelled cells) were used in combination with double-stained samples (AnnexinV-FITC/PI-labelled cells) for compensation controls (compensation matrix, FITC-PI 7.3% and PI-FITC 0.067%; automatic software compensation). Quadrant statistics revealed the fraction of vital and apoptotic cells for each sample (the early and late apoptotic cells were counted as one fraction of dead / apoptotic cells). All the experiments were performed in triplicate.

### Aneuploidy assay

To quantify the fraction of aneuploid cells, the treated cells were harvested, washed once with PBS, fixed overnight with 70% methanol, washed again, and resuspended in PBS containing 0.01 mg/ml RNase (Sigma Bio-Sciences, Deisenhofen, Germany). The cells were incubated at 37 °C for 30 min. The cell preparation was completed by the addition of 4’,6-diamidino-2-phenylindol (DAPI, Sigma Bio-Sciences) at a final concentration of 1 μg/ml. The DAPI fluorescence signal, as measured by flow cytometry, was plotted on a linear scale, and the ploidy analysis was performed using the multi-cycle algorithm of the ModFit LT 3.2 software (Verity Software House, Topsham, ME, USA), with cell doublet, aggregate, and debris discrimination via pulse processing.

### Western blotting

The cells were lysed in lysis buffer (New England Biolabs, Frankfurt, Germany) supplemented with 1 mM PMSF (Roth, Karlsruhe, Germany). Equal amounts of protein (15 μg) were separated by SDS-PAGE and transferred to Immobilon PVDF membranes (Millipore, Schwalbach, Germany). The blocking of nonspecific binding sites was performed using 5% (w/v) non-fat dry milk in TBST. The membranes were then incubated with primary antibodies diluted in 5% non-fat dry milk/ TBST for 12-14 hours at 4 °C. HRP-conjugated immunoglobulins (diluted 1:5000 in 5% non-fat dry milk/TBST) were used as the secondary antibodies and incubated for 1 h at room temperature. Immunoreactivity was visualised using the Pierce ECL Western Blotting Substrate (Thermo Scientific) and exposure to high-performance chemiluminescence film (G&E Healthcare, Freiburg, Germany).

### Statistical analyses

For interpretation of the immunohistochemical and PCR-RFLP data, statistical analyses were performed using SPSS software (version 18/19, IBM). A significance level of 5% was determined for all the statistical tests. First, the expression profiles of each marker in the different subgroups were compared (tumour versus normal tissue) using a nonparametric test (Wilcoxon-Mann-Whitney-Test). The overall survival was analysed by Kaplan-Meier curves and a log rank test.

Statistical analyses of the results from the *in vitro* experiments were performed using the Prism Graph Pad 5.0 software. Results with p-values < 0.05 were considered significant.

## SUPPLEMENTARY MATERIAL AND FIGURES


