# Supplementary material for: The response of head and neck squamous cell carcinoma to cetuximab treatment depends on Aurora kinase A polymorphism
Source: Oncotarget. 2014 Jun 18;5(14):5428–38. doi: 10.18632/oncotarget.2117 (PMC4170642; doi:10.18632/oncotarget.2117)
Supplement: Supplementary file 1 [file oncotarget-05-5428-s001.pdf]

The response of head and neck squamous cell carcinoma to cetuximab treatment depends on Aurora kinase A polymorphism

Supplementary Material

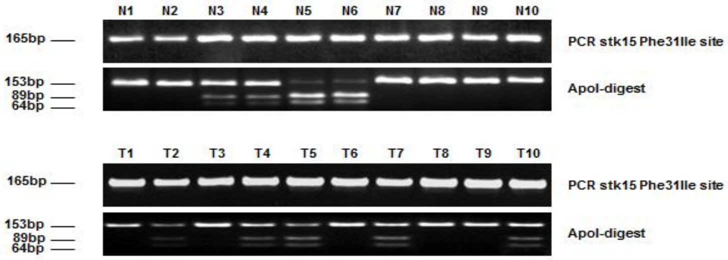

	Phe/Phe	Phe/Ile	Ile/Ile
normal tissue (n = 64)	43 (67%)	21 (33%)	0 (0%)
non neoplastic tissue (n=41)	26 (63%)	15 (37%)	0 (0%)
tumor tissue (n = 116)	56 (48%)	57 (49%)	3 (3%)

Suppl. 1: The distribution of AurkA/STK15 codon 91 homo- and heterozygosity was determined in normal, non-neoplastic, and tumour tissues.

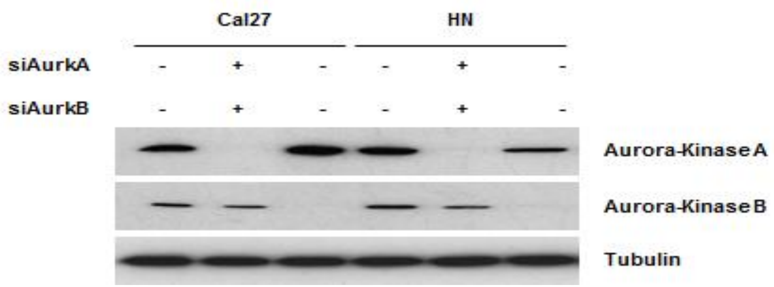

Suppl. 2: The siRNA-mediated knockdown of Aurora kinases A and B was highly effective, as determined by the protein expression levels of the respective kinase.

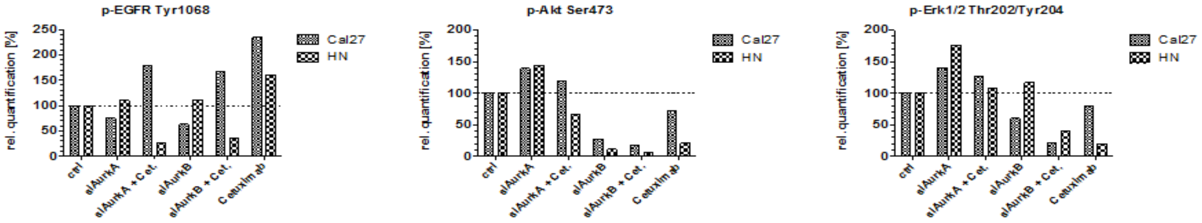

Suppl. 3: Shown is the relative quantification of western blot analyses. The EGFR, Akt, and Erk1/2 expression levels were not affected by any of the treatments. Cetuximab caused a marked increase in the phosphorylation of its target in the Cal27 but not the HN cells. Akt phosphorylation decreased after AurkB knockdown in both cell lines. Erk1/2 phosphorylation was decreased after AurkB knockdown in the Cal27 cells, in both the presence and absence of cetuximab.
